# Summative peer marking?

**DOI:** 10.1007/s40037-016-0257-5

**Published:** 2016-03-08

**Authors:** Lotte O’Neill, Anne Mette Morcke

**Affiliations:** Centre for Health Sciences Education (CESU), INCUBA Science Park, Aarhus University, Aarhus N, Denmark

In this issue, an interesting paper by Steverding et al. examines whether peer-rater bias (e.g. ‘friendly marking’) can be minimized to increase the reliability of summative peer marking [1]. Their intervention consisted of the introduction of a risk of receiving a ‘penalty’ in the form of a lower final grade for the peer markers, if their marks were inconsistent with the marks given by an experienced pair of examiners. In other words, peer-rater bias had consequences in the intervention group, but not in the control group. Contrary to their expectations, the results indicated that the intervention peer group were in fact more generous markers compared with the control group peers. In addition, they found that the top-performing students seemed to be particularly affected by the harsher marking in the control group. However, overall they still found high correlations between peer and examiner marks for both groups. We commend Steverding et al. for taking an interest in questions of the validity and reliability of peer assessment, because this topic is important irrespective of whether peer assessment is used summatively or formatively [2]. In addition, this paper sparked some thoughts on what is reasonable to expect when it comes to the agreement between peer and examiner marks (or novice and expert marks), and on the use of summative peer assessments.

A decade ago, a larger meta-analysis also revealed that peer marks tend to agree well with teacher marks, in particular if a global judgment was made and if it was based on well-understood assessment criteria [3]. The authors also found that agreement tended to be higher if academic processes and products were rated rather than professional practice. They stressed that student familiarity with and ownership of the assessment criteria enhance the validity of peer assessment. Others have also pointed out that a discrepancy between student and teacher understanding of the assessment criteria would give rise to inconsistencies in their marking when compared, and also that the lack of sufficiently broad background knowledge (expertise) on the topic to be assessed, such as for example not having read the same references as the peers being assessed, can be a perceived challenge for peer markers [4]. If we look at the literature about self-assessment, we might find more clues, because it contains results regarding the limitations of students as raters. There have been several studies examining students’ and other subjects’ ability to self-assess in many different contexts, and they have all concluded that lower performers generally tend to over-rate their own performances (e.g. by as much as 30 %), while only a smaller group of top performers tend to under-rate their own performances slightly compared with expert judgments [5, 6]. So, are there any indications that this tendency to be biased can be remedied with rewards or penalties? Economic rewards for accurate self-assessments have been tested, but could not remedy discrepancies between perceived and actual performances [5, 7]. In other words: the ability to self-assess is probably dependent on our competence levels and the majority of us most likely fail to self-assess accurately, precisely because of our incompetence and the resulting blind angles it leaves us. Looking at peer assessment with this backdrop, it is perhaps not entirely unreasonable to think that the level of one’s own subject expertise is indeed also important for accurate judgments of others’ performances. Therefore, it is probably only natural for students (novices) to not always be in complete agreement with the expert marker, even though they may be trying their very best to be. It seems to us that this discrepancy between student and teacher marks constitutes a window of opportunity for learning, if sufficient time is taken to explore disagreements, experts and novices together. High levels of agreement between peer and expert marks probably indicate that the teacher has had some success in transferring ownership of the assessment criteria to the students. However, such ownership usually comes at a price. It has, for example, been reported that even when a scoring rubric is co-created with students, teachers cannot expect that students know how to apply it independently. Even with such a level of ownership of the assessment criteria, students need specific explanations and also to practice mock critiques in plenum under the guidance of the teacher [2].

We agree with Steverding et al. that much of the literature on peer assessment revolves around its use for formative purposes as an important tool in ‘assessment for learning’ [1]. Some authors seem to think little of purely summative uses of peer feedback. Andrade, for example, commented on the use of scoring rubrics used in peer assessment [2]:

Students are not always good at peer- and self-assessment at first, even with a rubric in hand. At their worst, peer assessments can be cruel or disorienting….Rubrics used only to assign final grades represent not only a missed opportunity to teach but also a regrettable instance of the teacher as-sole-judge-of-quality model that puts our students in a position of mindlessness and powerlessness.

Along the same lines Lindblom-Ylänne et al. acknowledge that peer assessment can be either summative or formative [4], but at the same time state that ‘peer assessment should be formative in nature in order to enhance learning [8, 9], because summative peer assessment can undermine cooperation between students [10].’ However, in our experiences as teachers in medical education, students actually often express the need for both formative and summative types of feedback. So while many medical and other health science curricula may indeed suffer from too little focus on formative feedback, we also need to know whether and what summative feedback adds to formative feedback. Thinking back on some of the most severely struggling students we have met, the one thing they seemed to have in common was the inability to realistically judge their own performances in the exams, even in situations where continuous formative feedback had been given to them on their performances during the semester. They appeared unable to connect the dots between formative assessments and feedback and subsequent summative assessment results in time on their own. If we want to give these students maximal chances, we may need to supply them with the full feedback package (formative and summative assessments) as early and as repeatedly as possible within the educational framework we operate in. At the other end of the performance spectrum there are ambitious, competitive and bright students, who we also find request the summative aspects of assessment, possibly for them to feed or sustain their already high levels of self-efficacy and motivation, which we think are also legitimate and beneficial needs. Such a high level of formative and summative assessments would require an extended use of peer feedback in our setting. We hope that the study by Steverding et al. in this issue will inspire readers to explore further how to best use peer assessment in medical education, and whether there may be positive interactions with careful combinations of formative and summative assessments, which are also sufficiently feasible in practice.
